# Mesenchymal stem/stromal cells-derived IL-6 promotes nasopharyngeal carcinoma growth and resistance to cisplatin via upregulating CD73 expression

**DOI:** 10.7150/jca.37932

**Published:** 2020-02-03

**Authors:** Jincheng Zeng, Shasha Chen, Caihong Li, Ziyu Ye, Bihua Lin, Yanfang Liang, Bin Wang, Yan Ma, Xingxing Chai, Xin Zhang, Keyuan Zhou, Qunzhou Zhang, Haitao Zhang

**Affiliations:** 1 Guangdong Provincial Key Laboratory of Medical Molecular Diagnostics, Dongguan Key Laboratory of Medical Bioactive Molecular Developmental and Translational Research, Guangdong Medical University, Dongguan 523808, China; 2 Department of Biochemistry and Molecular Biology, Guangdong Medical University, Zhanjiang, China.; 3 Department of Oral and Maxillofacial Surgery and Pharmacology, University of Pennsylvania School of Dental Medicine, Philadelphia 19104, USA; 4 Department of Pathology, Dongguan Hospital Affiliated to Jinan University, The Fifth People's Hospital of Dongguan, Dongguan 523905, China; 5 Laboratory Animal Center, Guangdong Medical University, Zhanjiang, 524023 China; 6 Clinical Experimental Center, Jiangmen Central Hospital, Affiliated Jiangmen Hospital of Sun Yat-sen University, Jiangmen, 529030, China

**Keywords:** nasopharyngeal carcinoma, mesenchymal stem/stromal cells, tumor microenvironment, IL-6, CD73

## Abstract

Previous studies have implicated the important role of mesenchymal stem/stromal cells (MSCs) within tumor microenvironment (TME) in the pathogenesis and progression of nasopharyngeal carcinoma (NPC), but the potential mechanisms are still unclear. Herein, we showed that an elevated IL-6 level was positively correlated with elevated expression of CD73 in TME of NPC. NPC specimens with an IL-6^high^CD73^high^ phenotype showed higher expression levels of gp80, gp130, p-STAT3, MMP-9 and α-SMA, and clinically, a poorer prognosis than those with an IL-6^low^CD73^low^ phenotype. We found that stimulation with conditioned media derived from IL-6 gene knocked out MSC (MSC^IL6KO^-CM) down-regulated the expression of CD73, IL-6, gp80, p-STAT3, and proliferative cell nuclear antigen (PCNA) in CNE-2 NPC cells. Meanwhile, NPC cells co-cultured with MSC^IL6KO^-CM were more sensitive to cisplatin than those co-cultured with MSC-CM. Additionally, MSC-derived IL-6 transcriptionally upregulated CD73 expression *via* activating STAT3 signaling pathway in NPC cells. In summary, our findings suggest that MSCs promote NPC progression and chemoresistance by upregulation of CD73 expression *via* activating STAT3 signaling pathway.

## Introduction

Epidemiological trends over the past decade have shown that although the incidence of nasopharyngeal carcinoma (NPC) is gradually decreasing, NPC with high malignancy is still one of the common malignant tumors in Southeast Asia and North Africa 1, 2. So far, the 5-year survival rate of NPC is still less than 60%. The identified risk factors of NPC include Epstein-Barr virus (EBV) infection, genetic susceptibility, dietary habits, and environmental factors 1, 3, 4. However, the exact cause of NPC pathogenesis remains unclear. The tumor microenvironment (TME) is composed of cellular components such as infiltrating leucocytes, stromal cells and cancer cells, and non-cellular components such as extracellular matrix and various types of soluble biological factors or mediators. Recent studies have implicated the important role of TME, particularly, the function of stromal cells, in NPC pathogenesis and progression 4-7. However, the potential mechanisms by which mesenchymal stromal cells (MSC) in TME contribute to NPC pathogenesis and progression remain largely unknown. MSCs are pluripotent cells involved in a variety of physiological events, including maintenance of organogenesis and tissue homeostasis, and regeneration and repair of tissues. Tumors can be considered "wounds that never heal" and, in response to cues from a tumor, MSCs are continuously recruited to and become integral components of the tumor microenvironment. MSCs are also involved in the regulation of tumor development, in which they are increasingly recognized to play a complex role. Therefore, MSCs can directly affect tumor cells through secreted mediators and cell-cell interactions, and have an active role in tumour initiation, promotion, progression and metastasis by regulating innate and adaptive immune responses.

A wealthy body of evidence has shown that the IL-6/JAK/STAT3 signaling pathway is aberrantly activated in many types of cancer and associated with a poor prognosis 8. In TME, IL-6 could be produced by inflammatory cells 9, cancer cells 10, and MSCs 10-12, while hyperactivation of IL-6/JAK/STAT3 signaling pathways within a TME contributes to aggressiveness and progression of cancer through multifaced mechanisms. It can not only act intrinsically on tumor cells to promote cell proliferation, survival, epithelial-mesenchymal transition (EMT), cancer stem cell (CSC) properties, migration, invasion, and metastasis 12-16, but also act extrinsically on other type of cells within the complex TME to sustain a pro-tumor microenvironment by supporting angiogenesis and tumor evasion of immune surveillance 14. Noteworthy, IL-6 has also been shown to play an indispensable role in impeding tumor growth by mobilizing anti-tumor T cell immunity 14, 17. Previous studies have shown that extracellular adenosine acts as a potent immunosuppressive “halo” surrounding the tumor to interfere with anti-tumor immunity 18, 19. Extracellular adenosine is generated by a glycophosphatidylinositol-anchored receptor CD73, also known as ecto-5'-nucleotidase (NT5E) expressed on both tumor cells and stromal cells. Herein, we explored the relationship between IL-6 and CD73 in the TME of NPC, especially the potential contribution of MSC-derived IL-6 and CD73 expression to tumor growth and chemotherapeutic resistance in NPC.

## Results

### Elevated expression of IL-6 and CD73 in NPC

**Figure 1:**
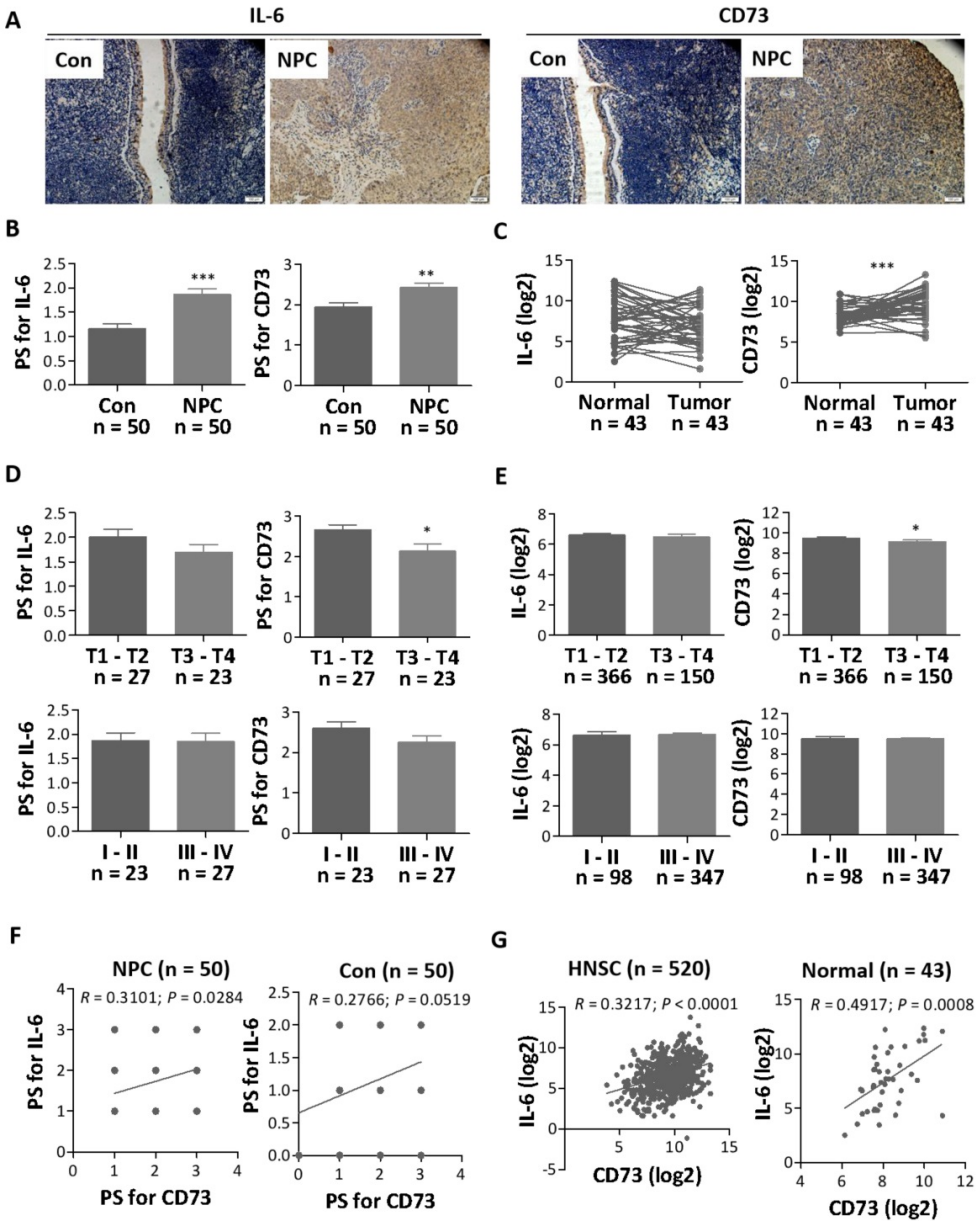
** The expression of IL-6 and CD73 in NPC. a.** Representative images for the IHC staining of IL-6 and CD73 in NPC and normal tissues. **b.** The relative expression levels of IL-6 and CD73 were analyzed by pathological score (PS) in all tissues. **c** The expression of IL-6 and CD73 in NPC and normal tissues were analyzed by HNSC RNA expression profile datasets from TCGA. **d-e** The differences in IL-6 and CD73 expression in different stages of NPC sections were analyzed based on PS** (d)** and TCGA datasets **(e)**.** f-g** Results from the Spearman correlation analysis of IL-6 with CD73 in all tissues based on PS** (f)** and TCGA datasets** (g)**. *, *P* < 0.05; **, *P* < 0.01; ***,* P* < 0.001.

Initially, we analyzed the expression profile of IL-6 in both NPC and normal control nasopharyngeal tissues by IHC. Our results showed that IL-6 was highly expressed in NPC tissues (n = 50) as compared with control nasopharyngeal tissues (n = 50) (Figure 1A and 1B). In addition, we also analyzed high throughput HNSC RNA expression profile datasets from The Cancer Genome Atlas (TCGA), but there was no significant difference in *IL6* mRNA expression between HNSC tissues and adjacent normal tissues (Figure 1C). Further correlative analyses showed that IL-6 expression was not strongly related to the patient's pathological stage and histological grade (Figure 1D and 1E).

Since IL-6 is a pleiotropic cytokine and plays a role in immune regulation of the tumor microenvironment^20^, we then explored the potential link between IL-6 expression and the CD73-adenosine axis, one of the key metabolic pathways or immune checkpoints that regulate tumor immunity^21^, ^22^. Our results showed that CD73, an adenosine-producing enzyme, was upregulated in NPC tissues as compared with control nasopharyngeal tissues and adjacent normal tissues (Figure 1A - 1C). In particular, CD73 expression was significantly higher in histological grade T1-T2 patients than in T3-T4 patients (Figure 1D and 1E). Then, we used protein chip to detect CD73 protein in four matched NPC tissues and paracancerous tissues. The results showed that CD73 protein was indeed highly expressed in NPC tissues (Figure S1). It's worthy to note that the expression of IL-6 was positively correlated with CD73 expression, especially in NPC tissues, at both protein (Figure 1F) and mRNA levels (Figure 1G). These studies suggest that IL-6 might be involved in regulating the expression of CD73 and the crosstalk between the two pathways may play a role in NPC progression.

### NPC patients with IL-6^high^CD73^high^ phenotype showed higher expressions of gp80, gp130, p-STAT3, MMP-9 and α-SMA, and a poorer prognosis than patients with IL-6^low^CD73^low^ phenotype

**Figure 2:**
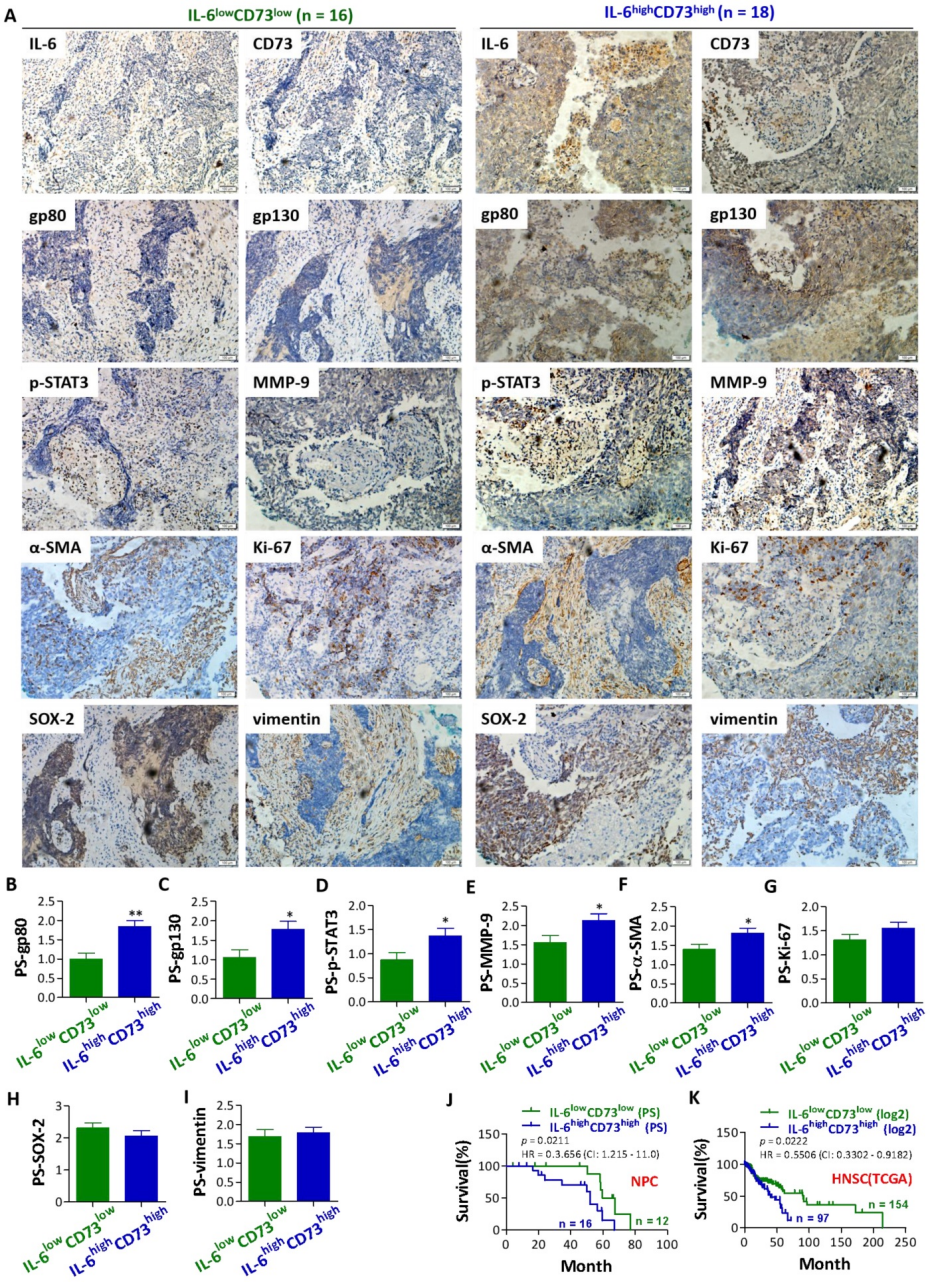
** Comparison of gp80, gp130, p-STAT3, MMP-9, α-SMA, Ki-67, SOX-2, and vimentin expression and prognosis between NPC patients with IL-6^high^CD73^high^ phenotype and IL-6^low^CD73^low^ phenotype. a.** Representative images for the IHC staining of gp80, gp130, p-STAT3, MMP-9, α-SMA, Ki-67, SOX-2, and vimentin in NPC patients with IL-6^high^CD73^high^ phenotype and IL-6^low^CD73^low^ phenotype. **b - i.** Bar graphic figures showing the relative expression levels, basing on pathological score (PS) of gp80 (**b**), gp130 (**c**), p-STAT3 (**d**), MMP-9 (**e**), α-SMA (**f**), Ki-67 (**g**), SOX-2 (**h**), and Vimentin (**i**) in NPC patients with IL-6^high^CD73^high^ phenotype and IL-6^low^CD73^low^ phenotype. **j - k.** Comparison of prognosis between NPC patients with IL-6^high^CD73^high^ phenotype and IL-6^low^CD73^low^ phenotype basing on PS (**j**) and TCGA datasets (**k**). *, *P* < 0.05; **, *P* < 0.01.

To further reveal the potential role of IL-6 and CD73 in NPC progression, patients with IL-6^high^CD73^high^ phenotype and IL-6^low^CD73^low^ phenotype were grouped according to the average expression of IL-6 and CD73. And then, the expression of gp80, gp130, p-STAT3, MMP-9, α-SMA, Ki-67, SOX-2, and vimentin in the above two phenotypes were comparatively analyzed. The results showed that gp80, gp130, p-STAT3, MMP-9 and α-SMA were highly expressed in patients with IL-6^high^CD73^high^ phenotype (Figure 2A-2G). IL-6 may act as an autocrine or paracrine growth factor for multiple cells. The binding of IL-6 to gp80 leads to an association and dimerization of gp130, followed by the rapid activetion of tyrosine kinases of the Jak and a subsequent activation of transcription factors of the STAT family. Hererin, our results show that the IL-6/STAT3 signal pathway in NPC tissue is abnormally activated. MMP-9 is an important cell invasion factor for NPC. High expression of MMP-9 is associated with lymph nodes metastasis and poor prognosis outcome. Our results also show that MMP-9 and α-SMA were high expressed on patients with IL-6^high^CD73^high^ phenotype. Significantly higher expression of α-SMA was observed in fibroblasts in NPC 23. Cancer-associated fibroblasts (CAFs) are major components of the surrounding stroma of carcinomas that emerge in the tumor microenvironment as a result of signals derived from the cancer cells. CAFs modulate growth factor signaling and extracellular matrix remodeling to regulate tumor metastasis. However, no significant differences were observed in the expression of Ki-67, SOX-2, and vimentin between patients with IL-6^high^CD73^high^ phenotype and patients IL-6^low^CD73^low^ phenotype (Figure 2A-2G). Interestingly, patients with IL-6^high^CD73^high^ phenotype showed a poorer prognosis than patients with IL-6^low^CD73^low^ phenotype based on both follow-up data and TCGA datasets of HNSC RNA expression (Figure 2J and 2K). These results further suggest that the correlation between IL-6 and CD73 pathways plays a role in NPC progression and prognosis.

### MSC-derived IL-6 activates STAT3 signaling pathway, induces CD73 expression, and promotes NPC tumor growth

**Figure 3:**
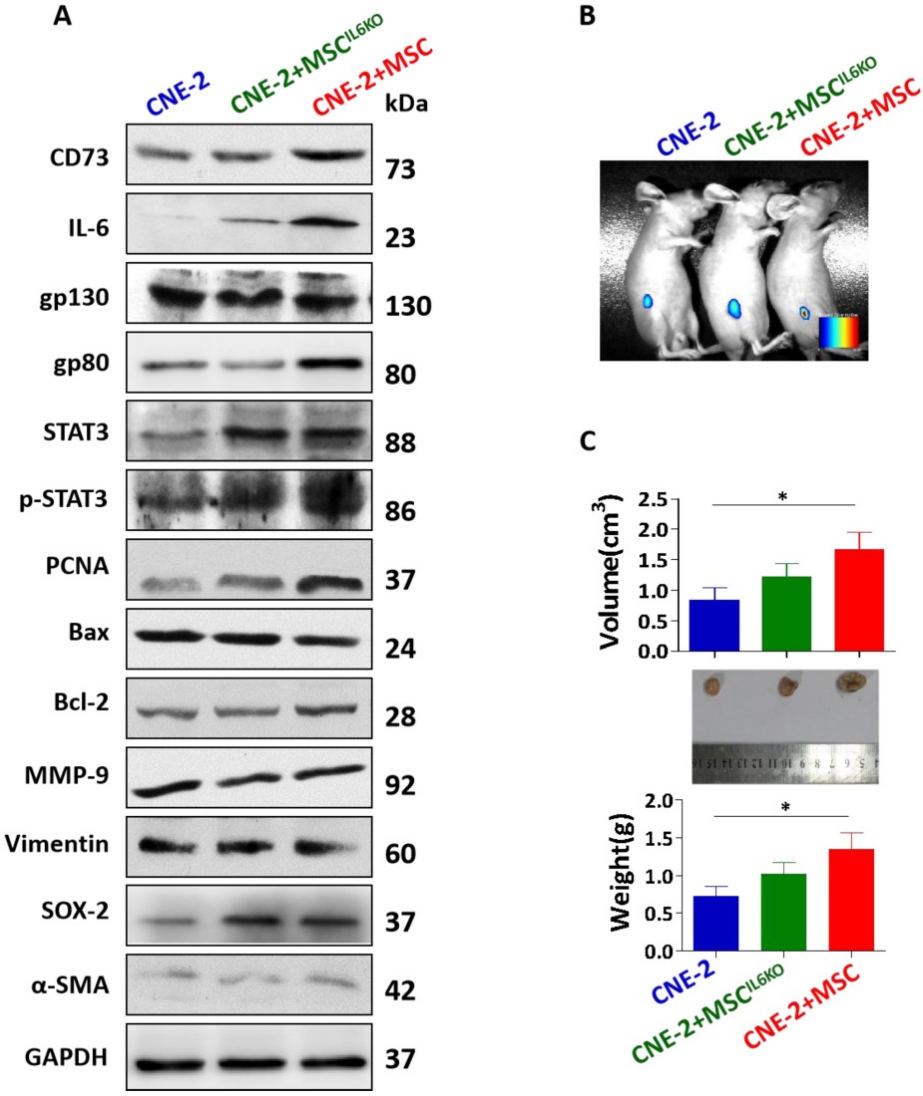
** MSC-derived IL-6 induces CD73 expression, activates STAT3 signaling pathway and promotes tumor growth. a.** The expression of CD73, IL-6, gp130, gp80, STAT3, p-STAT3, PCNA, Bax, Bcl-2, MMP-9, Vimentin, SOX-2, α-SMA were detected by western blot analysis. Luciferase activity imaging of tumor-bearing mice (**b**). Mice were euthanized and tumor volumes and weight were measured (**c**). *, *P* < 0.05; *P*=0.0675 (tumor volume: MSC^IL6KO^ *vs* MSC); *P*=0.0960 (tumor weight: MSC^IL6KO^ *vs* MSC)

We then explored the effect of IL-6 secreted by MSC on NPC cancer cells. Our results indicated that co-culture with BMSCs increased the proliferation rate of NPC cell lines, such as CNE-1 and CNE-2 (Figure S2). Moreover, BMSCs secreted an abundant level of IL-6 in the supernatants either cultured alone or co-cultured with NPC cells (Figure S3). Then, CNE-2 NPC cells were incubated with conditioned media (CM) derived from MSCs (MSC-CM) or MSC^IL6KO^ (MSC^IL6KO^-CM). The results showed that incubation with MSC^IL6KO^-CM down-regulated the expression of CD73, IL-6, gp80, p-STAT3, and PCNA in CNE-2 NPC cells (Figure 3A). However, there were no significant differences in the expression of gp130, STAT3, Bax, Bcl-2, MMP-9, vimentin, SOX-2, and α-SMA in CNE-2 NPC cells cultured with MSC^IL6KO^-CM or MSC-CM (Figure 3A). *In vivo* studies showed that co-transplantation of NPC cells with MSCs formed xenografted tumors that were significantly larger and heavier than those derived from transplanted NPC cells alone (*P*<0.05), suggesting the potent pro-tumorigenic activity of MSCs. However, co-transplantation of MSC^IL6KO^ showed moderately reduced pro-tumorigenic activity as evidenced by xenografted tumor formation with relatively smaller volume and less tumor weight as compared to those derived from co-transplantation of NPCs with MSCs (Figure 3B-3C). These results suggest that MSC-derived IL-6 contributes, at least in part, to NPC tumor growth *in vivo* possibly by mediating aberrant STAT3 activation and increased CD73 expression in TME.

### Exogenous and MSC-derived IL-6 promotes resistance of NPC cells to cisplatin

**Figure 4:**
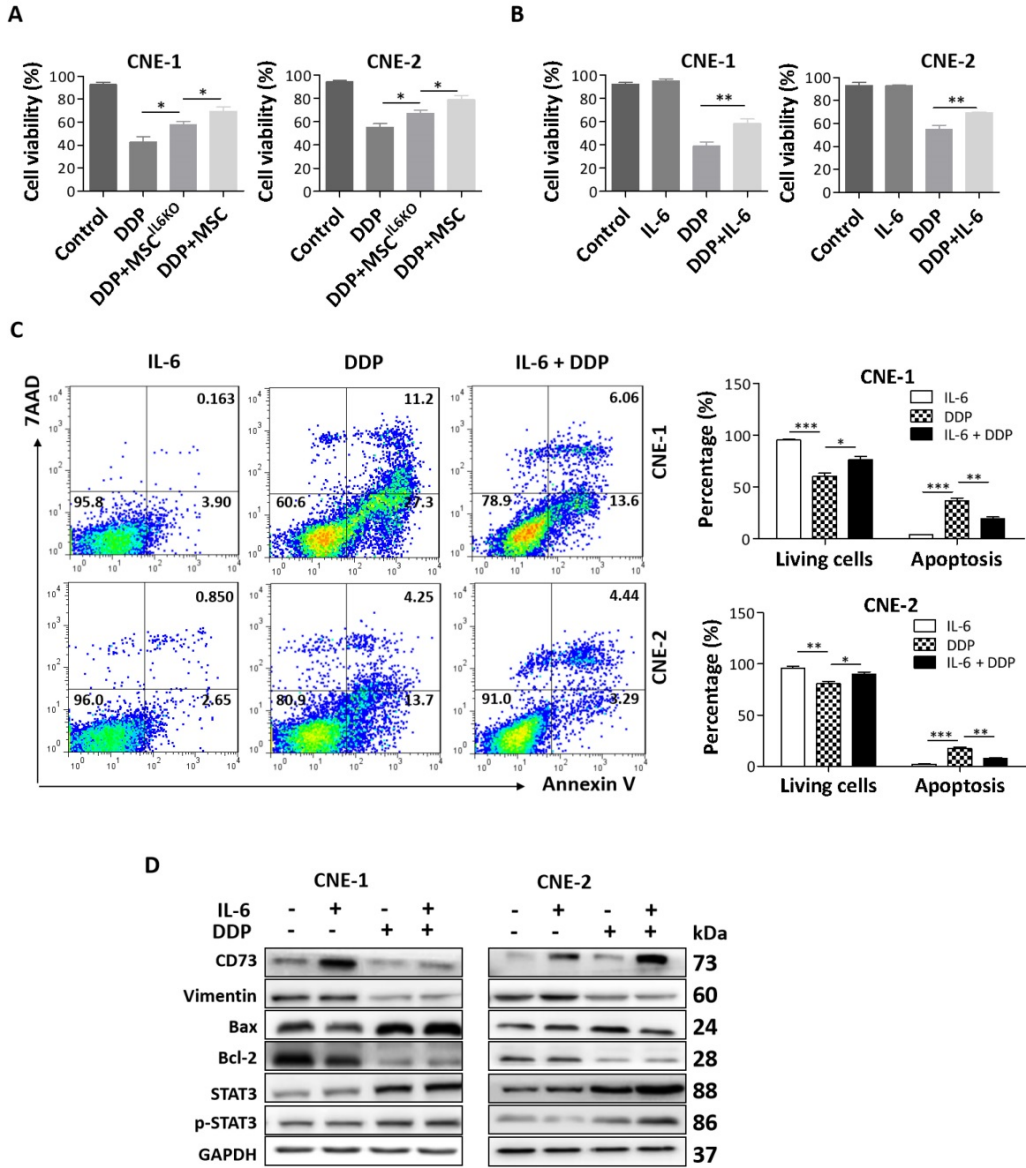
** MSC-derived IL-6 promotes NPC cells resistance to cisplatin via inducing CD73 expression. a.** Cell viability of the indicated cells under treatment of MSC^IL6KO^ or MSC-conditioned medium and 20 µM DDP. **b.** Cell viability of the indicated cells under treatment of 10 ng/mL IL-6 and 20 µM DDP. **c.** Annexin V-FITC/PI staining of the indicated cells under treatment of 10 ng/mL IL-6 and 20 µM DDP. **d.** Western blotting analysis of CD73, Vimentin, Bax, Bcl-2, STAT3, p-STAT3 in the indicated cells under treatment of 10 ng/mL IL-6 and 20 µM DDP. *, *P* < 0.05; **, *P* < 0.01.

Previous studies have shown that IL-6 enhances resistance of a variety of cancers to cisplatin (DDP) treatment *via* activating STAT3 pathway 29-35. Herein, we also investigated the effects of MSC-derived IL-6 on chemosensitivity of NPC cells to cisplatin. Our results showed that culture with MSC-CM obviously reduced DPP-induced loss of cell viability in both CNE-1 and CNE-2 cells, whereby such protective effects were significantly attenuated when NPC cells were cultured with MSC^IL6KO^-CM (Figure 4A). Similarly, exogenous IL-6 also obviously reduced DDP-induced loss of cell viability in both CNE-1 and CNE-2 cells (Figure 4B). Flow cytometric and Western blot analysis further showed that exogenous IL-6 not only enhanced the cell survivability but also significantly reduced total apoptosis induced by DDP in NPC cells (Figure 4C and 4D). Additionally, our results showed that IL-6-mediated increase in cell viability and inhibition of apoptosis in DPP-treated NPC cells correlated with STAT3 activation and increase of CD73 expression (Figure 4D).

### IL-6/STAT3 transcriptionally activates the promoter activity of *NET5* gene

**Figure 5:**
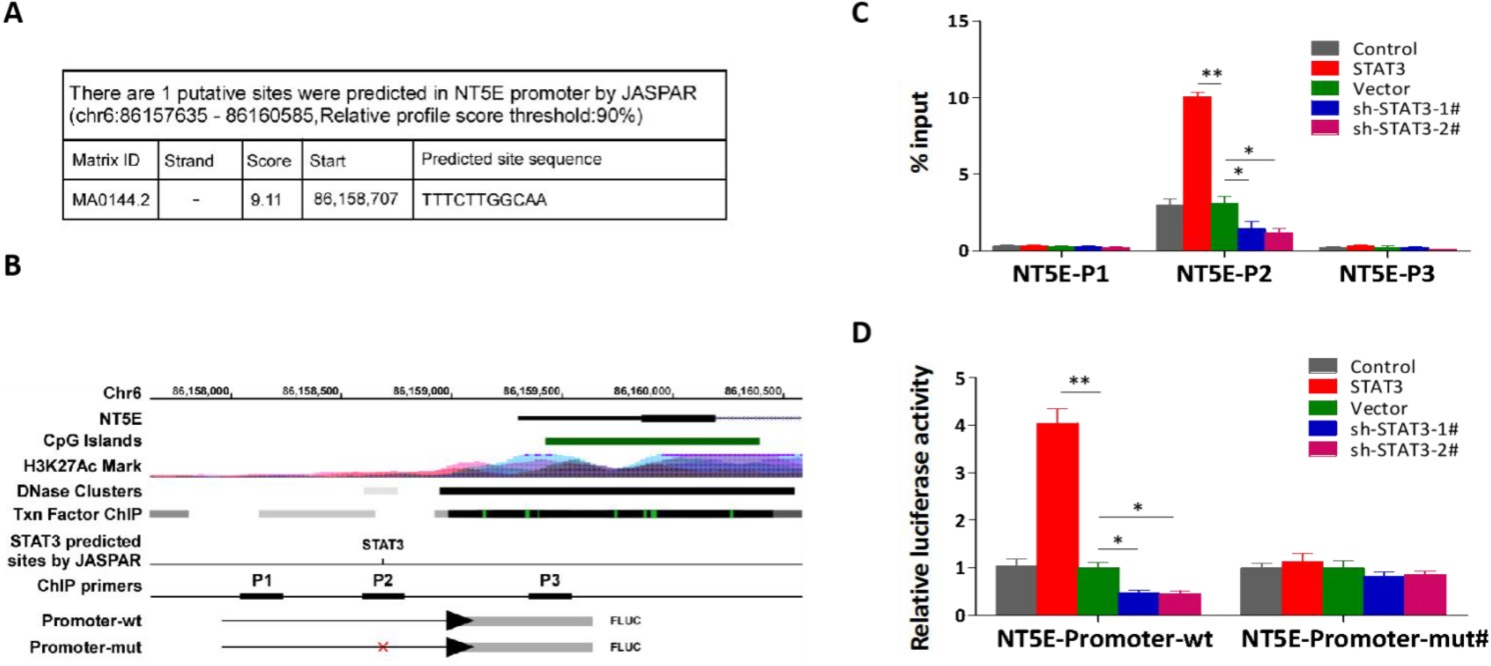
** STAT3 transcriptionally activating CD73. a.** The putative binding sites of STAT3 in *NT5E* (encoding CD73 protein) promoters by JASPAR (http://jaspar.genereg.net/). **b.** Schematic representation of the promoter regions of *NT5E* with the putative STAT3 binding sites through UCSC table browser (https://genome.ucsc.edu/cgi-bin/hgTables). **c.** Analysis of *NT5E* promoters physically associated with STAT3 by using ChIP assay in the indicated CNE-2 cells. **d** Relative luciferase activity of the indicated promoter vectors in the indicated CNE-2 cells.

Through analyzing JASPAR, we found one STAT3-binding motifs inside the putative promoter region of *NET5* (coding protein CD73) (Figure 5A). The UCSC bioinformatics identified three potential binding sites of STAT3 in the promoter region of *NET5* (Figure 5B). A ChIP assay indicated that STAT3 could bind to the P2 binding sites in the promoter region of *NET5* in NPC cells (Figure 5C). Furthermore, an enhancement of the *NET5* promoter luciferase activity was observed on upregulation of STAT3 in NPC cells. Conversely, downregulation of *NET5* significantly reduced the luciferase activity (Figure 5D). These findings suggest that IL-6/STAT3 upregulates CD73 expression through the binding of activated STAT3 to its promoter.

## Discussion

The TME is a dynamic milieu consisting of various cell types such as endothelial cells, fibroblasts, infiltrating leucocytes, and MSCs, and influences therapeutic responses and the clinical outcome. Microenvironment-mediated tumor growth and progression and drug resistance can be induced by soluble factors secreted by tumor or stromal cells. In particular, several lines of evidence have shown that elevated IL-6 and CD73 expressions within TME were positively correlated with the progression of various types of malignancies 14, 15, 18-20. To the best of our knowledge, this is the first study to show that IL-6 level was positively correlated with CD73 expression in TME of NPC, while patients with a IL-6^high^CD73^high^ phenotype showed a higher EMT score and poorer prognosis than patients with a IL-6^low^CD73^low^ phenotype. These findings support the notion that the cross-talk between IL-6 and CD73 pathways may play a role in the progression and prognosis of NPC.

As early as 1999, Huang et al reported that IL-6 was highly expressed in NPC biopsies by RT-PCR 24. Subsequently, Chow et al showed that increase of serum IL-6 was correlated with an advanced disease stage and a poorer prognosis for NPC patients 25. Moreover, serum IL-6 was decreased in NPC patients with effective therapies 26. In TME of NPC, IL-6 was expressed mainly in inflammatory cells 9, cancer cells 10, and MSCs 10-12. In several malignancies, multipotent MSCs, either tissue-specific resident MSCs or those recruited by cancer cells from bone marrow to tumor sites, contribute to tumor growth and progression 27-29. A growing body of evidence has demonstrated that tumor cells crosstalk with MSCs *via* a large panel of paracrine signaling factors, including various growth factors, cytokines, chemokines, extracellular vesicles, and many other mediators, which drive the EMT process, CSC formation, metastasis, and resistance to chemo- and radiation-therapies 29. Previously, several studies have implicated the potential role of IL-6 in the pathogenesis and progression of NPC. For instance, IL-6 has been shown to promote proliferation and invasion of NPC cells through STAT3 signaling 12, 30. Sun et al reported that IL-6 promoted migration and invasion of HNE1 and CNE1-LMP1 NPC cells by upregulating MMP-2 and MMP-9 expression 31. Chew et al also found that treatment with IL-6 enhanced MMP-9 production in TW01 and TW01-LMP1 NPC cells 32. In the present study, results from cytokine antibody microarray showed that co-culture of MSCs with NPCs can promote the secretion of a large number of cytokines such as VEGF, IL-6, and G-CSF from MSCs (Figure S3). Interestingly, our results showed that IL-6 secreted by MSCs leads to STAT3 activation and upregulation of CD73 expression in cancer cells, promoting xenograft NPC growth and resistance to cisplatin treatment. It is worth noting that MSC^IL6KO^ does not fully restore susceptibility to cisplatin, suggesting that IL-6 is not the only factor that reverses cisplatin resistance. Indeed, Roodhart et al.found that MSCs induce resistance to chemotherapy through the release of two distinct platinum-induced polyunsaturated fatty acids (PIFAs), 12-oxo-5,8,10-heptadecatrienoic acid (KHT) and hexadeca-4,7,10,13-tetraenoic acid (16:4(n-3)) 33. In addition, miR-1180 from bone marrow-derived MSC can induce glycolysis and cisplatin resistance in ovarian cancer cells 34. Jia et al. found that 14-3-3ζ which contained in human umbilical cord MSC also can enhance autophagy to alleviate cisplatin-induced acute kidney injury 35.

Notably, our results show that both IL-6 and CD73 has no significant relationship with histopathological staging of NPC. However, when we combined the expression of IL-6 and CD73 to study the relationship between different IL-6 and CD73 expression types and disease progression, we found that NPC patients with IL-6^high^CD73^high^ phenotype showed higher expressions of gp80, gp130, and p-STAT3, lower expressions of MMP-9 and α-SMA, and a poorer prognosis than patients with IL-6^low^CD73^low^ phenotype. Indeed, local immune contexture do not reflect the pathology-related practices for specific tumor types,combined into an 'Immunoscore', which has been shown to complement the prognostic ability of the TNM staging for carcinomas^36^. Taken together, these findings support that elevated IL-6 in TME may play an important role in pathogenesis and progression of NPC.

**Figure 6:**
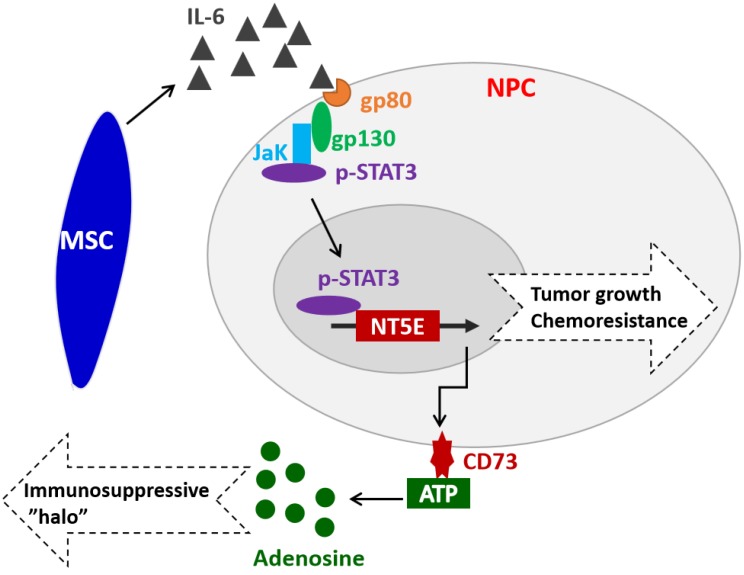
Hypothetical model illustrating that MSC-derived IL-6 upregulates CD73, promotes tumor growth and chemoresistance in NPC.

Extracellular ATP plays a critical role in coordinating appropriate inflammatory/immune responses in various pathological processes by acting on various types of immune cells 37. The ecto-enzymes CD73 can dephosphorylate extracellular ATP to adenosine, which has potent immunosuppressive and anti-inflammatory functions, thus contributing to the establishment of tumor immunosuppressive microenvironment (TIME) 21, 22. Recent studies have shown that CD73 expression and adenosine generation by MSCs and effector T cells in TME were up-regulated by TGF-β 38-40. Herein, we found that CD73 expression in NPC cells was up-regulated by extracellular IL-6 or MSC-derived IL-6 *via* activating STAT3, which subsequently binds to the promotor of *NET5* gene and transcriptionally activates its expression. Interestingly, extracellular adenosine can also stimulate various types of cells, such as cholangiocyte, human dermal microvascular endothelial cells, dendritic cells, airway epithelia, pituitary folliculostellate cells, and astrocytes, to secrete IL-6 *via* the A2bAR 41-46. In summary, our findings have revealed the link between IL-6/STAT3 pathway and CD73-adenosine axis, which might play an important role in tumor growth and chemoresistance in NPC (Figure 6). Further studies are warranted to elucidate the complicated interactions or feedback regulatory loops between these two pathways and their contribution to pathogenesis, immune surveillance, and progression of NPC. Currently, some targeted drugs have been developed for both IL-6 and CD73. However, their medicinal effects in tumors are still controversial. In this study, we further clarified the relationship between IL-6 and CD73 in NPC carcinoma, which will provide guidance for the combination therapy using IL-6 or CD73 antibody drugs.

## Materials and methods

### Patients and tumor tissues

Fifty cases NPC patients and fifty cases non-NPC patients were obtained at the Department of Otorhinolaryngology, Affiliated Hospital of Guangdong Medical University (Guangdong, China) and Affiliated Jiangmen Hospital of Sun Yat-sen University (Guangdong, China) between December 2011 and January 2013. Informed consent and ethical approval were granted by the patients and the two hospitals' Institutional Research Ethics Committee, respectively. Patients were diagnosed based on clinical and pathological evidence, and the patient's lesion was surgically removed without any chemotherapy or radiation therapy. The cancer tissue was quickly frozen after it is removed. The pathological characteristics of NPC patients were detailed in Table S1. The proportions of tumor vs. non-tumor in Hematoxylin & Eosin (HE) staining tissues were evaluated by three independent professional pathologists.

### Cell line and cell culture

The human nasopharyngeal carcinoma cell lines CNE-1, CNE-2 were obtained from Dongguan Key Laboratory of Medical Bioactive Molecular Developmental and Translational Research, Guangdong Medical University, China, and cultured in DMEM medium (Life Technologies, Carlsbad, CA, US) supplemented with penicillin G (100 U/ml), streptomycin (100 mg/ml) and 10% fetal bovine serum (FBS, Life Technologies). Human bone marrow mesenchymal stem cells (BM-MSC) with detailing differentiation and phenotypic characteristics 47 were obtained from Center for Stem Cell Biology and Tissue Engineering, Sun Yat-sen University, China. BM-MSC^IL6KO^ was established using IL-6 CRISPR/Cas9 KO Plasmid according to our previous report 48. BM-MSC and BM-MSC^IL6KO^ were cultured in L-DMEM medium supplemented with 10% FBS. All cells were incubated at 37 °C in a humidified atmosphere with 5% CO_2_ and were routinely sub-cultured using 0.25% (w/v) trypsin (without EDTA) solution.

### Immunohistochemistry (IHC)

As our previously reported 49, 50, the tumor tissue were fixed in formalin, embedded in paraffin, sectioned and then heat-immobilized, quenched the endogenous peroxidase activity and retrieved antigenic epitopes. For immunostaining, primary antibodies against IL-6 (sc-28343, Santa Cruz), CD73 (PA5-29750, Thermo-Fisher), gp80 (IL-6Rα, sc-373708, Santa Cruz), gp130 (sc-655, Santa Cruz), p-STAT3 (sc-8001-R, Santa Cruz), SOX-2 (66411-1-Ig, Proteintech), MMP-9 (sc-393859, Santa Cruz), Ki-67 (27309-1-AP, Proteintech), Vimentin (sc-373717, Santa Cruz) and α-SMA (55135-1-AP, Proteintech) were incubated for 30 min at room temperature, followed by HRP-conjugated secondary detection antibody and diaminobenzidine (DAB).

All sections were assessed by two pathologists in a blinded fashion to the clinical status of the patients. The immunoreactive area for selected protein was scored as 0(0~5%), 1 (5%~25%), 2 (26~50%), 3 (51~75%) or 4 (> 76%). According to the dyeing strength, 0 point for colorless, 1 point for pale yellow, 2 points for tan color, and 3 points for brown tan color. Results are scored by multiplying the score of immunoreactive area and dyeing strength. Finally, based on the results, the expression level and pathological score (PS) were defined. 0 was negative expression (PS = 0), 1 to 4 was low expression (PS = 1), and 5 to 8 was medium expression (PS = 2), 9 to 12 was high expression (PS = 3).

### Preparation of MSC-conditioned medium

BM-MSC cells (1.0 × 10^6^) were seeded in 35-mm plates and cultured in L-DMEM with 15% FBS. The following day, the media was removed and the cells were washed with PBS, re-incubated with fresh medium for 48h. Then, BM-MSC-conditioned medium (MSC-CM) and BM-MSC^IL6KO^-conditioned medium (MSC^IL6KO^-CM) were collected respectively and centrifuged at room temperature to remove possible cell debris.

### Cytokine antibody microarray

The cell culture supernatants were collected by centrifugation at 12000 rpm for 5 min, filtered with a 0.22 µm disposable filter, temporarily stored at -80 °C, and then sent in dry ice to Guangzhou GeneSeed Biotech for cytokine antibody microarray (RayBiotech AAH-CYT-1000) analysis.

### CCK-8 assay

To investigate the effect of MSC-CM on cell sensitivity to cisplatin (DDP), CNE-1 and CNE-2 NPC cells (2 × 10^3^) were seeded into 96 well plates and treated with cisplatin (DDP, 20 µM) alone or in combination with either MSC-CM, MSC^IL6KO^-CM, or IL-6 (10 ng/mL, Sigma). Cell viability was determined using CCK-8 kit (Beyotime, China) and the OD values at 450 nm were measured using an ELISA reader (BioTek, Winooski, VT, USA) according to the manufacturer's instructions.

### Western blotting analysis

Nuclear/cytoplasmic fractions were separated by using Cell Fractionation Kit (Cell Signaling Technology, USA) according to the manufacturer's instructions, and the whole cell lysates were extracted using RIPA Buffer (Cell Signaling Technology). Western blot was performed according to a standard method as described previously 49. Protein bands were visualized using ECL reagents (Pierce, USA). Antibodies against IL-6 (sc-28343, Santa Cruz), CD73 (PA5-29750, Thermo-Fisher), gp80 (IL-6Rα, sc-373708, Santa Cruz), gp130 (sc-655, Santa Cruz), p-STAT3 (sc-8001-R, Santa Cruz), SOX-2 (66411-1-Ig, Proteintech), MMP-9 (sc-393859, Santa Cruz), Ki-67 (27309-1-AP, Proteintech), Vimentin (sc-373717, Santa Cruz), α-SMA (55135-1-AP, Proteintech), STAT3 (sc-8019, Santa Cruz), PCNA (10205-2-AP, Proteintech), Bax (50599-2-Ig, Proteintech) and Bcl-2 (12789-1-AP, Proteintech) were used. The membranes were stripped and reprobed with an anti-GAPDH antibody (sc-47724, Santa Cruz) as the loading control.

### Tumor xenografts

Experimental procedures were approved by the Institutional Animal Care and Use Committee of Guangdong Medical University. 6-week-old BALB/c-nu mice were randomly divided into three groups (n = 5 per group): CNE-2 cells (1 × 10^7^) alone, CNE-2 cells (1 × 10^7^) in combination with BM-MSC (1 × 10^7^) , and CNE-2 cells (1 × 10^7^) in combination with BM-MSC^IL6KO^ (1 × 10^7^). Cells in 100 µL of PBS were inoculated subcutaneously into the inguinal folds of the nude mice. Tumor volume was determined weekly using an external caliper and calculated using the equation (L × W^2^)/2. On day 33, tumors were detected by an IVIS imagining system (Caliper, USA). Then animals were euthanized, and tumors were excised, weighed, and stored in liquid nitrogen for further analysis.

### Cell apoptosis assay

Cell apoptosis was evaluated by flow cytometric analysis following staining with the APC Annexin V Apoptosis Detection Kit with 7-AAD (BioLegend, CA, USA) according to manufacturer's protocol as described before 50, 51. The BD FACS Calibur II (San Jose, CA, USA) platform was used to acquire data. All Data were analyzed by using Flowjo.7.6.1 software (Treestar, USA) as instructed.

### Transfection

Plasmid, siRNA for STAT3 was obtained from Ribobio (Guangzhou, China). Transfection of siRNAs and plasmids was performed using Lipofectamine 3000 (Life Technologies) according to the manufacturer's instructions.

### Real-time polymerase chain reaction (RT-PCR)

RNA extraction, reverse transcription, cDNA amplification, and real-time PCR were performed according to our previous report 51. Primers for NT5E and GAPDH were synthesized and purified by RiboBio (Guangzhou, China), whereby the expression of GAPDH mRNA was used as endogenous controls. The primer sequences were provided in Table S2. Relative fold expressions were calculated with the comparative threshold cycle (2^-ΔΔCt^) method according to the previous study.

### Chromatin immunoprecipitation (Chip)

As previously reported^52^, cross-linking was performed with formaldehyde (Merck) at a final concentration of 1% and terminated 5 min later by the addition of glycine at a final concentration of 0.125 M. Cells were harvested with SDS buffer, pelleted by centrifugation, and resuspended in IP buffer. Chromatin was sheered by sonication (HTU SONI 130; Heinemann) to generate DNA fragments ranged from 300bp to 900bp, followed by preclearing and incubation with antibodies against STAT3 or IgG control for 16 h. Immunoprecipitated DNA was purified and then analyzed by qPCR. The primers for ChIP were provided in Table S2.

### Luciferase assay

Cells (3 × 10^4^) were seeded in 24-well plates and cultured for 24 h, and the luciferase reporter assay was performed as previously described 51, 52. Cells were transfected with 100 ng NT5E promoter reporter luciferase plasmid plus 5 ng pRL-TK Renilla plasmid (Promega) using Lipofectamine 3000 (Invitrogen) according to the manufacturer's recommendation. Luciferase and Renilla signals were measured 36 h after transfection using a Dual Luciferase Reporter Assay Kit (Promega) according to the manufacturer's protocol.

### Statistical analysis

All values are presented as mean ± standard error of the mean (SEM). Significant differences were determined using GraphPad 5.0 software (GraphPad Software Inc., San Diego, CA, USA). Student's t-test was employed to determine statistical differences between two groups. One-way ANOVA was used to determine statistical differences between multiple testing. Spearman and Pearson correlation was used to measure the degree of dependency between variables. A value of *P* < 0.05 was deemed significant, and values of *P* < 0.01 and *P* < 0.001 were considered as highly significant. All the experiments were repeated three times.

## Supplementary Material

Supplementary figures and table.
