# Mesenchymal stem/stromal cells-derived IL-6 promotes nasopharyngeal carcinoma growth and resistance to cisplatin via upregulating CD73 expression: Erratum

**DOI:** 10.7150/jca.104693

**Published:** 2024-10-15

**Authors:** Jincheng Zeng, Shasha Chen, Caihong Li, Ziyu Ye, Bihua Lin, Yanfang Liang, Bin Wang, Yan Ma, Xingxing Chai, Xin Zhang, Keyuan Zhou, Qunzhou Zhang, Haitao Zhang

**Affiliations:** 1 Guangdong Provincial Key Laboratory of Medical Molecular Diagnostics, Dongguan Key Laboratory of Medical Bioactive Molecular Developmental and Translational Research, Guangdong Medical University, Dongguan 523808, China; 2 Department of Biochemistry and Molecular Biology, Guangdong Medical University, Zhanjiang, China.; 3 Department of Oral and Maxillofacial Surgery and Pharmacology, University of Pennsylvania School of Dental Medicine, Philadelphia 19104, USA; 4 Department of Pathology, Dongguan Hospital Affiliated to Jinan University, The Fifth People's Hospital of Dongguan, Dongguan 523905, China; 5 Laboratory Animal Center, Guangdong Medical University, Zhanjiang, 524023 China; 6 Clinical Experimental Center, Jiangmen Central Hospital, Affiliated Jiangmen Hospital of Sun Yat-sen University, Jiangmen, 529030, China

Following the publication of the above article, an interested reader drew to the authors' attention that, for the Figure 2A the representative images selected for the 'α-SMA' in NPC patients with IL-6^low^ CD73^low^phenotype and 'vimentin' in NPC patients with IL-6^high^ CD73^high^ phenotype were found to be overlapping. After having consulted our original data files, we realized that the Figure 2A had been inadvertently assembled incorrectly. We wish to emphasize that the corrections made to the Figure 2A do not affect the overall conclusions reported in the paper. We really regret that the error went unnoticed prior to the publication of this article, and we are grateful to the Editor of the Journal of Cancer for allowing us the opportunity to publish this erratum. All the authors agree to the publication of this erratum, and Zhang's group also apologizes for any inconvenience this has caused to the reviewers of this article and readers of the journal.

The revised version of Figure 2A, presenting the correct image for the 'vimentin' in NPC patients with IL-6^high^ CD73^high^ phenotype, is shown below.

## Figures and Tables

**Figure 2:**
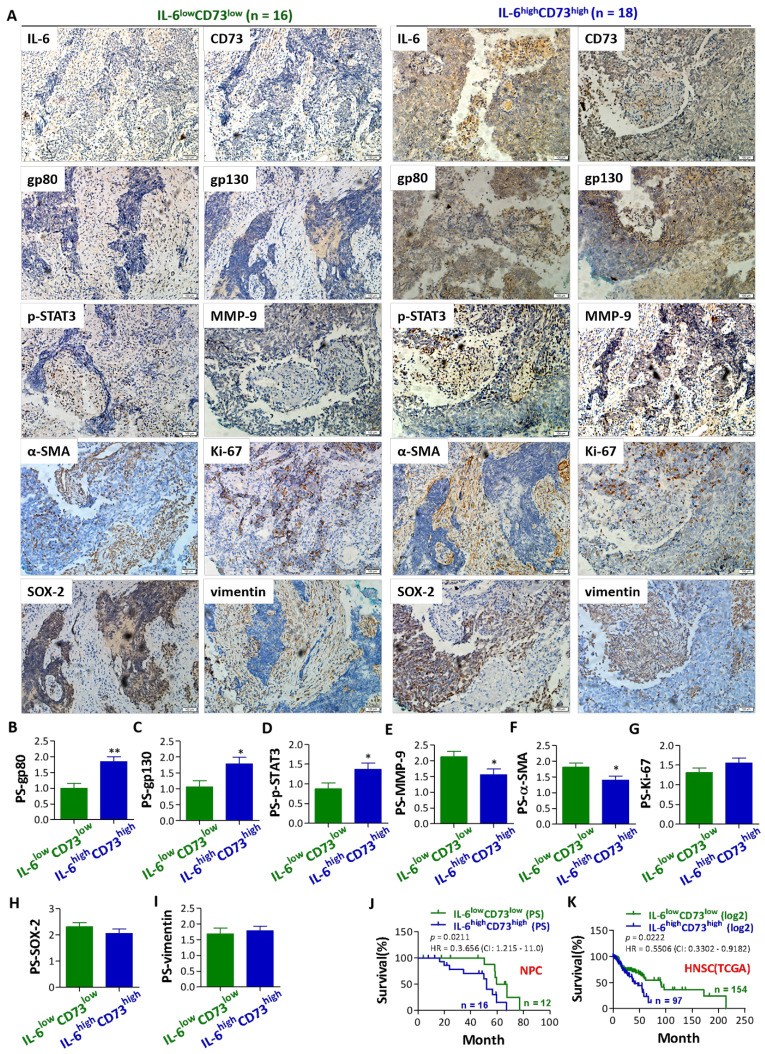
Correct image.

