# Supplementary material for: Mesenchymal stem/stromal cells-derived IL-6 promotes nasopharyngeal carcinoma growth and resistance to cisplatin via upregulating CD73 expression
Source: J Cancer. 2020 Feb 3;11(8):2068–79. doi: 10.7150/jca.37932 (PMC7052921; doi:10.7150/jca.37932)
Supplement: Supplementary file 1 — Supplementary figures and table. [file jcav11p2068s1.pdf]

Supplementary Material

Table S1. NPC patients' characteristics

Parameters		n
Gender		
	Female	23
	Male	27
Age (years)		
	<50	20
	≥50	30
Smoking history		
	Yes	31
	No	19
Clinical stage		
	I	12
	II	11
	III	16
	IV	11
T stage		
	T1	14
	T2	13
	T3	14
	T4	9

Table S2. A list of primers used in the reactions

NT5E(CD73) promoter-F	5'-GTACCCACAGATACCAGGCAATG-3'
NT5E(CD73) promoter-R	5'-GTTTGTGGTTGCGCTAGCTC-3'
NT5E(CD73) promoter-mut-F	5'-ATGGCCCCCAGGCCTGGATGATTTACAATCTCCGAACCTCAG-3'
NT5E(CD73) promoter-mut-R	5'-AATCATCCAGGCCTGGGGGCCATGCAGTTTGGGAG-3'
NT5E(CD73)-P1-F	5'-CTTGGCCTAAGGACAGACTGG 3'
NT5E(CD73)-P1-R	5'-AGTTCAAGCTTCGCAAACTGG-3'
NT5E(CD73) -P2-F	5'-CATTAGAGGGTGTCCCAGAAGC-3'
NT5E(CD73)-P2-R	5'-TGACCTGAGGTTCGGAGATTG-3'
NT5E(CD73)-P3-F	5'-CTCAGCTCGCTCATCTTTCTTC-3'
NT5E(CD73)-P3-R	5'- CTCTGAGTCTCGCCTCATTCG-3'
GAPDH-F	5'-ATTCCACCCATGGCAAATTC-3'
GAPDH-R	5'-TGGGATTTCCATTGATGACAAG-3'

Supplementary Figures legends

Figure S1. Protein chip detection in four matched NPC tissues and paracancerous tissues

Figure S2. MSC promote NPC cell lines CNE-1 and CNE-2 cell proliferation

Cell viability of CNE-1 (A) and CNE-2 (B) under treatment of MSC-conditioned medium. *, $P < 0.05$; **, $P < 0.01$; * **, $P < 0.001$.

Figure S3. Detection of cytokine secreted by MSC via cytokine antibody array.

Detection of supernatant cytokine secreted by CNE-2, MSC, CNE-2 mixed MSC co-culture(1:1) (A). Differential cytokine analysis between CNE-2 mixed MSC co-culture(1:1) and CNE-2 (B). Differential cytokine analysis between CNE-2 mixed MSC co-culture(1:1) and MSC (C). Differential cytokine analysis between MSC and CNE-2 (D).

Supplementary Figures

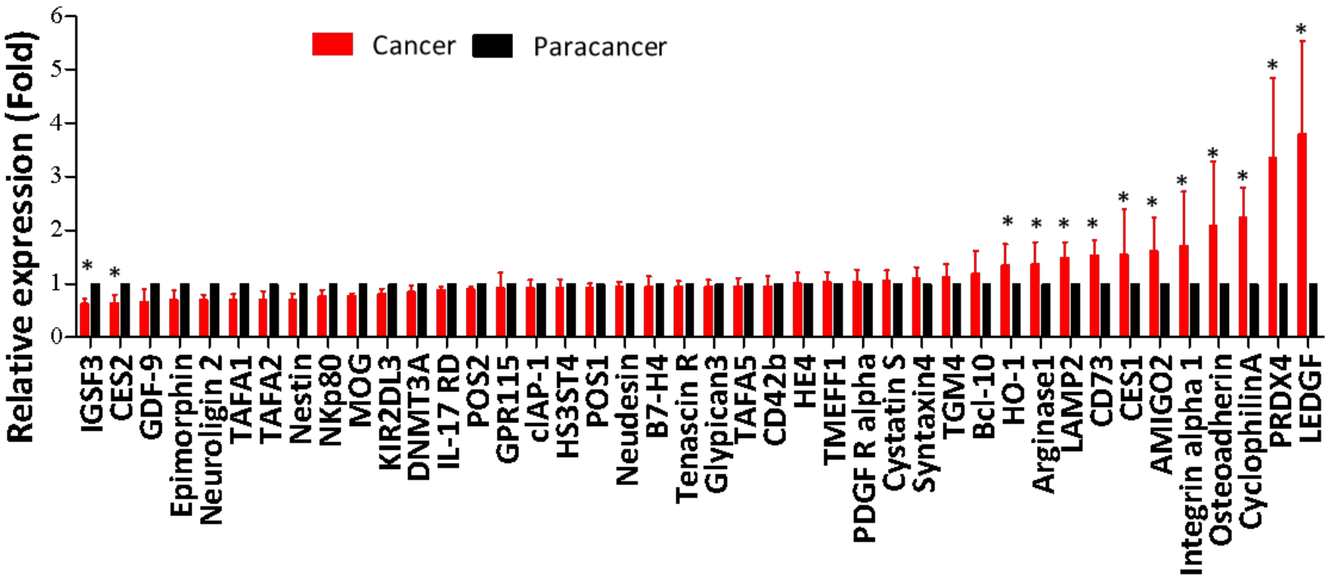

Figure S1. Protein chip detection in four matched NPC tissues and paracancerous tissues

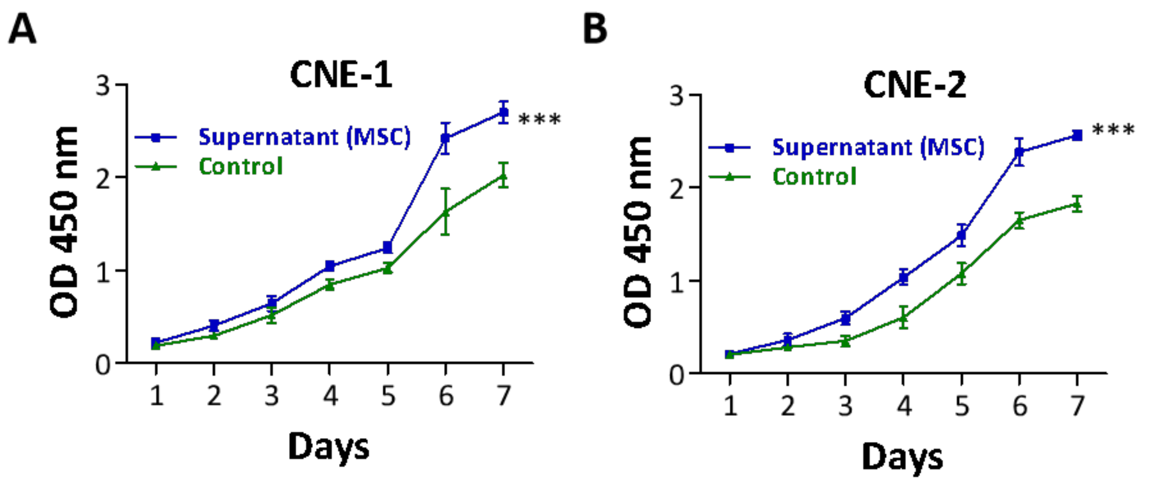

Figure S2. MSC promote NPC cell lines CNE-1 and CNE-2 cell proliferation

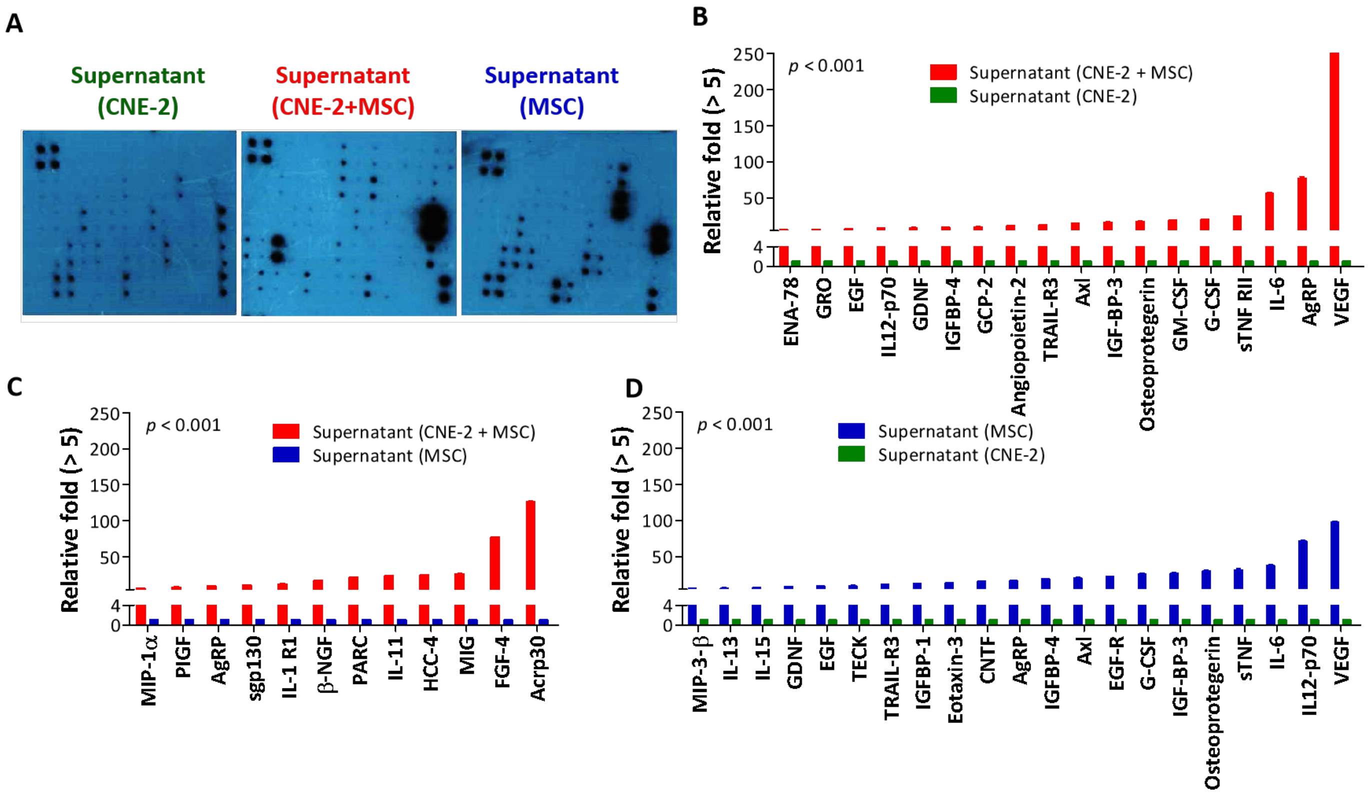

Figure S3. Detection of cytokine secreted by MSC via cytokine antibody array.
